# The concurrence of sexual violence and physical fighting among adolescent suicide ideators and the risk of attempted suicide

**DOI:** 10.1038/s41598-022-09387-3

**Published:** 2022-03-28

**Authors:** Xun Li, Shi-Ting Xiang, Jie Dong

**Affiliations:** 1 Pediatrics Research Institute of Hunan Province, Hunan Children’s Hospital, 86 Ziyuan Road, Changsha, China; 2 Department of Pediatric Intensive Care Unit, Hunan Children’s Hospital, 86 Ziyuan Road, Changsha, China

**Keywords:** Health care, Risk factors

## Abstract

Sexual violence and physical fighting are independent risk factors for suicidal behaviors among adolescents. However, whether the concurrence of these two risk factors increases the risk of suicidal behaviors among ideators, and by how much that risk increases are unknown. This study analyzed data from the 2019 National Youth Risk Behavior Surveys. Students who reported having seriously considered attempting suicide in the past year were included (n = 1755). The associations between physical fighting/sexual violence status and the risk of suicide attempt/plan/attempt requiring medical intervention were estimated. The concurrence of physical fighting and sexual violence substantially increased the relative risk of attempted suicide (adjusted relative risk, adRR = 1.99, 95% CI 1.72, 2.29) and attempted suicide requiring medical intervention (adRR = 4.07, 95% CI 2.84, 5.85; subgroup analyses: among women, adRR = 3.33, 95% CI 2.14, 5.17; among men, adRR = 6.25, 95% CI 3.32, 12.28). Among students who had experienced concurrent physical fighting and sexual violence, more suicide-attempt-associated health-risk behaviors were reported by men (median = 14) than women (median = 12) (*p* = 0.0023). The concurrence of physical fighting and sexual violence among adolescent suicide ideators was shown to be significantly associated with an increased risk of attempted suicide. A wide variety of health-risk behaviors were shown to cluster with the concurrence of physical fighting and sexual violence. This was especially the case among men, consistent with their higher rate of attempted suicide requiring medical intervention.

## Introduction

Suicide is one of the leading causes of death among adolescents worldwide^1^. Globally, suicide was the fourth leading cause of death among 15- to 19-year-olds in 2019^1^. A reduction of the suicide rate among adolescents is challenging but a prerequisite is a better understanding of suicidal behaviors in this age group. Previous studies showed that only a small subset of those with suicidal ideation will go on to attempt suicide, and even fewer will die by suicide^2^. For example, in the 2019 National Youth Risk Behavior Surveys (YRBS) from the United States, 18.8% of the investigated high school students reported having seriously considered suicide, 15.7% reported having made a suicide plan, 8.9% reported having attempted suicide, and 2.5% reported having made a suicide attempt that required medical treatment^3^. As suicidal ideation alone is not an effective predictor of attempted suicide, identifying the risk factors for suicidal behaviors among ideators is important for the development of intervention programs. Moreover, given that a history of attempted suicide is one of the most consistent risk factors for suicide^2^, and a history of attempted suicide is associated with long-standing psychosocial impairment^4,5^, investigations of the risk factors and behavior patterns associated with attempted suicide among adolescent suicide ideators may improve the health of adolescents and adults.

A wide range of risk factors associated with the transition from suicide ideator to attempter have been identified, ranging from biological to behavioral and sociological factors^2,6–8^. However, when considered individually, most of these risk factors are poorly predictive of suicidal behaviors^9–11^. As individuals who will die by suicide usually exhibit numerous risk factors, rather than a single risk factor in isolation, exploring the effects of combined risk factors might improve our understanding of pathways that result in attempted suicide as well as provide novel information allowing suicide attempters to be distinguished from suicide ideators^11^.

Experiences of sexual violence and physical fighting are independent risk factors for suicidal behaviors^12–15^. Their importance in the pathways of adolescent suicidal behaviors was highlighted in a recent study that used classification tree analysis to identify patterns of risk factors that differentiate suicide attempters from ideators among adolescents^11^. The best-performing tree included three classification variables: lifetime history of rape, past-year physical fighting, and heroin use. Compared with adolescents characterized by none of these three variables (rate of attempted suicide = 29%), the risk of being an attempter was higher among adolescents who had experienced past-year physical fighting and heroin use (78%), adolescents who had experienced past-year physical fighting but no heroin use (around 42%), and adolescents who had experienced rape (58%). Although the overall classification accuracy was modest (area under the curve: 0.65), the findings of that study suggested that the associations between sexual violence, physical fighting, and suicidal behaviors among adolescents merit further investigation.

In a preliminary study, we found an overlap between the population who had experienced sexual violence and the population who had reported past-year physical fighting among adolescents. According to the interpersonal theory of suicide, individuals with a suicidal desire can acquire suicide capability^2^, to which both sexual violence and physical fighting could contribute. However, whether the concurrence of these two risk factors further increases the risk of suicidal behaviors among ideators, and by how much that risk increases are unclear. Answering this question will not only help to identify high-risk populations among adolescents but it will also provide insights into how multiple factors are related to suicidal behaviors among adolescent ideators.

In studies of the epidemiology of suicidal behavior, one of the most consistent findings is a sex difference. Worldwide, men deaths by suicide outnumber those of women, although nonlethal suicide attempts by women outnumber those by men^16^. As pathways of suicidal behaviors may differ between the two sexes, we speculated that the effects of sexual violence and physical fighting on the risk of suicidal behaviors likewise differ.

In previous studies, sexual violence was shown to be associated with other health-risk behaviors, such as cigarette smoking and marijuana use^17^, which also served as risk factors for suicidal behaviors. Similar patterns were observed for physical fighting. For example, weapon-carrying is associated with an increased risk of physical fighting and suicidal behaviors^18,19^. According to problem behavior theory, in adolescents deprived of a healthy environment, health-risk and other problematic behaviors may cluster together^20^. Although the mechanisms underlying the interaction of multiple factors in suicide pathways are complex and difficult to conceptualize, based on the problem behavior theory, we propose that individuals exposed to concurrent physical fighting and sexual violence will have more health-risk behaviors than individuals with only one of these exposures and that they will, in turn, have a higher risk of suicidal behaviors.

Drawing from the finding of previous studies and from existing theories, we hypothesized that among adolescent suicide ideators: (1) the concurrence of sexual violence and physical fighting would be associated with an increased risk of suicidal behavior; (2) the strength of the association between concurrent physical fighting/sexual violence and suicidal behaviors would differ between men and women; (3) individuals who had experienced concurrent physical fighting and sexual violence would exhibit more health-risk behaviors, which in turn would be linked to more severe suicidal behaviors. To test our hypotheses, we used 2019 YRBS data from adolescents who had reported seriously considering attempting suicide in the past year. According to their replies to the queries on past-year physical fighting and sexual violence, the respondents were categorized into four groups: (1) no physical fighting and no sexual violence; (2) sexual violence without physical fighting; (3) physical fighting without sexual violence; and (4) physical fighting and sexual violence. First, the general characteristics of the respondents as well as the rates of suicide plan, attempted suicide, and attempted suicide requiring medical intervention were determined for each group, followed by the associations between physical fighting/sexual violence status and the risk of suicide plan/attempt/attempt requiring medical intervention. A subgroup analysis according to sex was also conducted. We then analyzed the distribution of other suicide-attempt-associated health-risk behaviors among the four groups and compared the cumulative number of risk behaviors among those groups and in men vs. women.

## Methods

### Data source

Publicly available data from the 2019 YRBS were analyzed. YRBS is a cross-sectional, school-based survey administered in public and private schools in the U.S. by the Centers for Disease Control. Details on this survey can be found at^21^. In the 2019, 181 schools were sampled, with students in grades 9 through 12 included in the sampling frame. The overall response rate (school response rate multiples the student response rate) was 60.3%^21^. The present study included students who reported having seriously considered attempting suicide within the past year (Question 26 in the questionnaire). Students who had seriously considered attempting suicide but had missing data for past-year suicide behaviors (skipped Question 28 for past-year attempted suicide) were excluded.

The national YRBS was approved by the Institutional Review Board (IRB) at the US Centers for Disease Control and Prevention. The participation of students in the YRBS is anonymous and voluntary. Before survey administration, informed consent of parents was obtained according to local parental permission procedures. During survey administration, students completed the self-administered questionnaire during one class period and recorded their responses directly on a computer-scannable booklet^21^. In this study, all YRBS data analyses methods were performed in accordance with the guidelines and regulations provided by the Centers for Disease Control and Prevention.

### Measures

The 2019 YRBS included four queries on suicide ideation and attempt: “Q26. During the past 12 months, did you ever seriously consider attempting suicide?” (answer: Yes/No); “Q27. During the past 12 months, did you make a plan about how you would attempt suicide?” (answer: Yes/No); “Q28. During the past 12 months, how many times did you actually attempt suicide? ” (answer: 0 times/1 time/2 or 3 times/4 or 5 times/6 or more times); and “Q29. If you attempted suicide during the past 12 months, did any attempt result in an injury, poisoning, or overdose that had to be treated by a doctor or nurse?” (answer: I did not attempt suicide during the past 12 months/Yes/No).

The main outcome variable was attempted suicide (Q28). This variable was analyzed as an ordinal categorical variable (times of attempted suicide: 0, 1, 2–3, 4–5, and ≥ 6), as well as a dichotomized variable, such that responses with one or more times of suicide attempts were coded as suicide attempt (yes) and responses with zero times of suicide attempts were coded as suicide attempt (no). The secondary outcome variables were planned suicide (Q27) and suicide attempt requiring medical intervention (Q29). Suicide attempt requiring medical intervention were dichotomized such that responses of “I did not attempt suicide during the past 12 months” and “No” were coded as no and responses of “Yes” was coded as yes.

Physical fighting was measured using the following items: “Q17. During the past 12 months, how many times were you in a physical fight?”; and “Q18. During the past 12 months, how many times were you in a physical fight on school property?”. A binary variable was created such that respondents who reported one or more times of physical fight and respondents who reported one or more times of physical fight on school property were coded as past-year physical fight (yes), and respondents reported neither of these experiences were coded as past-year physical fight (no). Sexual violence was measured using the following items: “Q19. Have you ever been physically forced to have sexual intercourse when you did not want to? ”; “Q20. During the past 12 months, how many times did anyone force you to do sexual things that you did not want to do? (Count such things as kissing, touching, or being physically forced to have sexual intercourse.)”; and “Q21. During the past 12 months, how many times did someone you were dating or going out with force you to do sexual things that you did not want to do? (Count such things as kissing, touching, or being physically forced to have sexual intercourse.) ”. A binary variable was created such that all respondents with forced sexual intercourse (Q19), all respondents with one or more times of sexual violence (Q20), and all respondents with one or more times of sexual dating violence (Q21) were coded as past-year sexual violence (yes), and respondents reported none of these experiences were coded as past-year sexual violence (no).

According to the status of past-year physical fighting and past-year sexual violence, the respondents were categorized into four groups: (1) physical fighting (no) and sexual violence (no) (physical fighting –/sexual violence –); (2) physical fighting (no) and sexual violence (yes) (physical fighting –/sexual violence +); (3) physical fighting (yes) and sexual violence (no) (physical fighting +/sexual violence –); and (4) physical fighting (yes) and sexual violence (yes) (physical fighting +/sexual violence +).

The associations between 59 risk behaviors investigated in the 2019 YRBS and the risk of suicide attempt were screened using a Chi-squared test or Fisher's exact test. The 42 risk behaviors found to be positively associated with the risk of attempted suicide (*p* < 0.05) were included in this study as suicide-attempt-associated health-risk behaviors (Supplementary Table 1).

### Statistical analysis

Categorical variables are presented as absolute values and percentages. Between-group comparisons were conducted for categorical variables using a Chi-squared test or Fisher's exact test, as appropriate, and for ordinal categorical variables using Wilcoxon rank sum test. All pairwise comparisons were conducted by applying a Bonferroni correction (αʹ = 0.05/number of comparison sets). Log-binomial regression was used to assess the associations between physical fighting/sexual violence status and suicidal behaviors (suicide plan, attempted suicide, and attempted suicide requiring medical intervention). The adjusted relative risk (adRR) and 95% CI were calculated and adjusted for the demographic variables found to be significantly associated with physical fighting/sexual violence status. Subgroup analyses of the associations between physical fighting/sexual violence status and suicidal behaviors were conducted according to sex. The attributable risk percent (AR%) was calculated as: (adRR-1)/adRR × 100%. Survey weights were not used because our research questions focused on the associations between physical fighting/sexual violence status and attempted suicide among adolescents who had seriously considered attempting suicide, rather than on an estimate of population prevalence. Missing data were not imputed. All tests were two-tailed, with a type 1 error rate fixed at 5%. All statistical analyses were performed using SAS 9.3 (SAS Institute, Inc., Cary, NC).

## Results

### Study groups and suicide behaviors

Among the 13,677 respondents in the 2019 YRBS, 2633 reported having seriously considered attempting suicide in the past year. The 538 respondents who skipped the query of suicide attempt, and the 340 respondents who skipped the queries for physical fighting and/or sexual violence were excluded from this study. Thus, the final analyses were based on 1755 respondents. This population was categorized into four groups according to physical fighting/sexual violence status: physical fighting –/sexual violence – (n = 827, 47.12%); physical fighting –/sexual violence + (n = 360, 20.51%); physical fighting +/sexual violence − (n = 318, 18.12%); and physical fighting +/sexual violence + (n = 250, 14.25%). Supplementary Fig. 1 shows the flow chart of the study population.

The demographic characteristics and the rates of past-year attempted suicide among four groups are presented in Table 1. Significant differences were observed between the (physical fighting –/sexual violence –) group and the other groups with respect to sex distribution, sexual orientation, grade, and the rates of suicide planning, attempt, and attempt requiring medical intervention (*p* < 0.05). The (physical fighting + /sexual violence +) group had the highest rates of suicide planning (75.5%), attempted suicide (63.6%), and attempted suicide requiring medical intervention (28.4%).

**Table 1:** Demographic characteristics and past-year suicide attempts among adolescents who reported having seriously considered attempting suicide during the past year, as reported in the 2019 YRBS.

Characteristics	All ^a^	Physical fighting/sexual violence groups						
		–/– (Group 1)	–/+ (Group 2)		+/– (Group 3)		+/+ (Group 4)	
		n (%)	n (%)	*p *^*b*^	n (%)	*p *^*b*^	n (%)	*p *^*b*^
Total	1755	827	360		318		250	
**Race**								
White	828 (48.4)	390 (48.3)	178 (50.4)	0.4265	142 (46.4)	0.4825	118 (48.2)	0.1639
Black	216 (12.6)	106 (13.1)	37 (10.5)		42 (13.7)		31 (12.7)	
Hispanic	122 (7.1)	68 (8.4)	24 (6.8)		19 (6.2)		11 (4.5)	
Other races/multiple races	546 (31.9)	244 (30.2)	114 (32.3)		103 (33.7)		85 (34.7)	
**Sex**								
Woman	1129 (65.1)	530 (64.8)	308 (86.3)	** < 0.0001**	123 (39.2)	** < 0.0001**	168 (68.9)	0.2409
Man	604 (34.9)	288 (35.2)	49 (13.7)		191 (60.8)		76 (31.1)	
**Sexual orientation**								
Heterosexual	1085 (63.7)	530 (65.2)	180 (52.0)	**0.0002**	238 (77.5)	**0.0007**	137 (57.6)	0.1398
Gay or lesbian	107 (6.3)	51 (6.3)	27 (7.8)		13 (4.2)		16 (6.7)	
Bisexual	387 (22.7)	168 (20.7)	109 (31.5)		45 (14.7)		65 (27.3)	
Not sure	125 (7.3)	64 (7.9)	30 (8.7)		11 (3.6)		20 (8.4)	
**Grade**								
9th grade	468 (26.9)	202 (24.6)	89 (24.9)	**0.0053**	104 (33.2)	**0.0068**	73 (29.3)	0.0721
10th grade	489 (28.1)	235 (28.6)	80 (22.3)		91 (29.1)		83 (33.3)	
11th grade	411 (23.6)	208 (25.3)	80 (22.3)		72 (23.0)		51 (20.5)	
12th grade	373 (21.4)	177 (21.5)	109 (30.4)		46 (14.7)		41 (16.5)	
Suicide plan	1143 (65.5)	503 (61.0)	251 (69.9)	**0.0035**	204 (64.6)	0.2740	185 (75.5)	** < 0.0001**
Attempted suicide	723 (41.2)	259 (31.3)	172 (47.8)	** < 0.0001**	133 (41.8)	**0.0008**	159 (63.6)	** < 0.0001**
Attempted suicide requiring medical intervention	171 (11.3)	49 (6.7)	36 (11.9)	**0.0054**	30 (10.5)	0.0436	56 (28.4)	** < 0.0001**

^a^The sum of each group do not always equal the total because of missing data.

^b^Compared with Group 1. Bold values were statistically significant after Bonferroni correction (αʹ = 0.05/3 = 0.0167).

### Frequency of physical fighting, sexual violence, and attempted suicide

**Figure 1:**
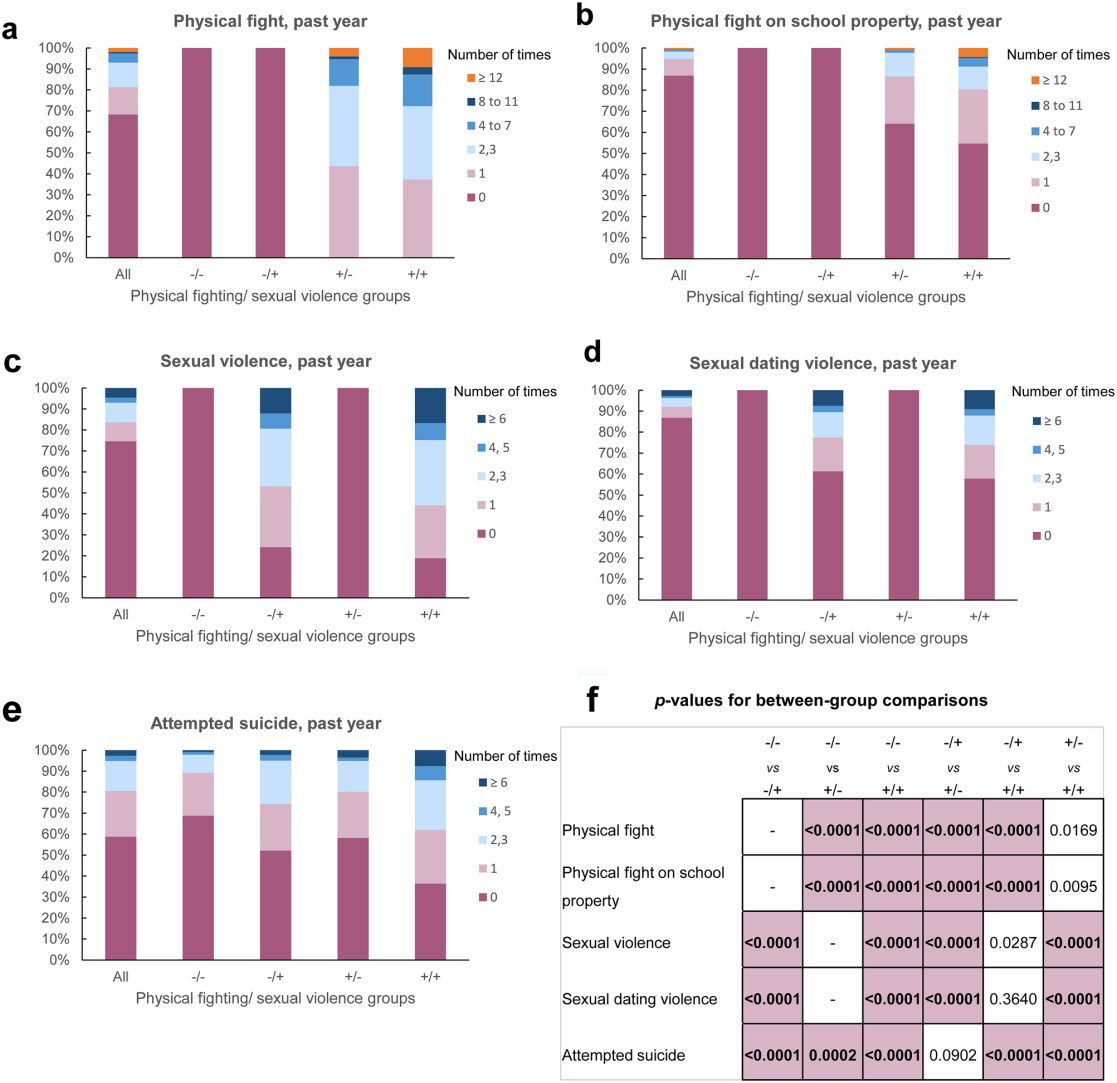
Number of times of physical fighting, sexual violence, and attempted suicide during the past year. (**a**) Physical fighting, (**b**) physical fighting on school property, (**c**) sexual violence, (**d**) sexual dating violence, and (**e**) attempted suicide. (**f**) *p* values for pair-wise between-group comparison of frequency distributions in a Wilcoxon rank sum test. Purple-shaded boxes indicate statistically significant associations determined after Bonferroni correction (αʹ = 0.05/6 = 0.0083).

Figure 1 shows the frequency of physical fighting, sexual violence, and attempted suicide during the past year. The distribution of attempted suicide did not significantly differ between the (physical fighting –/sexual violence +) and (physical fighting +/sexual violence –) groups, whereas attempted suicide was more frequent in the (physical fighting +/sexual violence +) group than in the other groups (*p* < 0.0001).

### Associations between physical fighting/sexual violence status and suicidal behaviors

Table 2 shows the association between physical fighting/sexual violence status and suicidal behaviors. Compared with the (physical fighting –/sexual violence –) group, the risks of attempted suicide and attempted suicide requiring medical intervention were significantly higher in the other three groups (all *p* < 0.05). The adRRs for attempted suicide and attempted suicide requiring medical intervention in the (physical fighting –/sexual violence +) and (physical fighting +/sexual violence –) groups ranged from 1.37 to 1.62, whereas the adRRs of the (physical fighting +/sexual violence +) group were even higher (attempted suicide, adRR = 1.99; attempted suicide requiring medical intervention, adRR = 4.07; all *p* < 0.05). Weak but significant associations were observed between the risk of suicide plan and the (physical fighting –/sexual violence +), as well as the (physical fighting +/sexual violence +) group (adRR = 1.11 and 1.21, respectively; all *p* < 0.05).

**Table 2:** Association between physical fighting/sexual violence status and suicidal behaviors.

Sex	Outcome	Physical fighting/sexual violence	Event %	adRR (95%CI) ^a^	*p*	AR%
All	Suicide plan	–/–	61	1 (Reference)		0
		–/+	69.9	**1.11 (1.01, 1.22)**	**0.0213**	10.11%
		+/–	64.6	1.09 (0.99, 1.21)	0.0805	8.59%
		+/+	75.5	**1.21 (1.1, 1.32)**	** < 0.0001**	17.32%
	Attempted suicide	–/–	31.3	1 (Reference)		0
		–/+	47.8	**1.51 (1.29, 1.75)**	** < 0.0001**	33.60%
		+/–	41.8	**1.37 (1.14, 1.63)**	**0.0005**	27.02%
		+/+	63.6	**1.99 (1.72, 2.29)**	** < 0.0001**	49.64%
	Attempted suicide requiring medical intervention	–/–	6.7	1 (Reference)		0
		–/+	11.9	**1.62 (1.04, 2.48)**	**0.028**	38.20%
		+/–	10.5	**1.6 (1, 2.5)**	**0.0443**	37.33%
		+/+	28.4	**4.07 (2.84, 5.85)**	** < 0.0001**	75.43%
Women	Suicide plan	–/–	63.8	1 (Reference)		0
		–/+	70.5	1.1 (1, 1.21)	0.057	9.06%
		+/–	66.4	1.06 (0.91, 1.2)	0.4453	5.29%
		+/+	76	**1.16 (1.04, 1.28)**	**0.0063**	13.67%
	Attempted suicide	–/–	30.9	1 (Reference)		0
		–/+	49	**1.63 (1.37, 1.94)**	** < 0.0001**	38.56%
		+/–	54.5	**1.75 (1.4, 2.15)**	** < 0.0001**	42.88%
		+/+	63.1	**1.98 (1.65, 2.36)**	** < 0.0001**	49.48%
	Attempted suicide requiring medical intervention	–/–	7.7	1 (Reference)		0.00%
		–/+	11.5	1.49 (0.92, 2.38)	0.0977	32.78%
		+/–	14.7	**1.92 (1.05, 3.31)**	**0.0243**	47.88%
		+/+	26.5	**3.33 (2.14, 5.17)**	** < 0.0001**	69.98%
Men	Suicide plan	–/–	55.4	1 (Reference)		0
		–/+	66.7	1.17 (0.9, 1.45)	0.184	14.81%
		+/–	62.6	1.16 (0.99, 1.36)	0.0617	13.88%
		+/+	74.3	**1.38 (1.15, 1.62)**	**0.0002**	27.48%
	Attempted suicide	–/–	31.9	1 (Reference)		0
		–/+	38.8	1.12 (0.69, 1.65)	0.6159	10.39%
		+/–	33.5	1.05 (0.8, 1.37)	0.7174	4.88%
		+/+	64.5	**2.04 (1.6, 2.59)**	** < 0.0001**	51.09%
	Attempted suicide requiring medical intervention	–/–	5.1	1 (Reference)		0
		–/+	15.8	2.18 (0.64, 5.77)	0.1504	54.12%
		+/–	7.5	1.55 (0.72, 3.33)	0.2531	35.58%
		+/+	32.8	**6.25 (3.32, 12.28)**	** < 0.0001**	84.00%

*adRR* adjusted relative risk, *AR%* attributable risk percent.

^a^For the analysis that included the whole study population, relative risks were adjusted for sex, sexual orientation, and grade. In the subgroup analysis by sex, relative risk were adjusted for sexual orientation and grade.

Significant values are in bold.

### Subgroup analysis according to sex

In the subgroup analysis (Table 2), men and women in the (physical fighting +/sexual violence +) group had the highest risk of suicide plan, attempted suicide, and attempted suicide requiring medical intervention (all *p* < 0.05). However, men and women differed with respect to the patterns of association. Among men, the associations were significant only in the (physical fighting +/sexual violence +) group. Among women, a higher risk of attempted suicide was observed for those in the (physical fighting –/sexual violence +) group (adRR = 1.63, 95% CI 1.37, 1.94) and the (physical fighting +/sexual violence –) group (adRR = 1.75, 95% CI 1.40, 2.15). Women in the (physical fighting +/sexual violence –) group also had an increased risk of attempted suicide requiring medical intervention (adRR = 1.92, 95% CI 1.05, 3.31). Strong associations were observed between the (physical fighting +/sexual violence +) and attempted suicide requiring medical intervention for both women (adRR = 3.33, 95% CI 2.14, 5.17) and men (adRR = 6.25, 95% CI 3.32, 12.28). The AR% for attempted suicide requiring medical intervention was 69.98% among women and 84.00% among men.

### Attempted-suicide-associated factors

Table 3 shows the distribution of 42 attempted-suicide-associated factors among the four groups. Compared with the (physical fighting –/sexual violence –) group, the other three groups had significantly higher rates of most of the investigated risk behaviors. The (physical fighting +/sexual violence +) group had the highest rates for all of the investigated risk behaviors. The risk behavior profiles were more similar between the (physical fighting +/sexual violence –) and (physical fighting –/sexual violence +) groups, with the exception of the rates of weapon carrying, physical dating violence, electronic bullying, initiation of alcohol use (≤ 12 years old), ever ecstasy use, ever sexual intercourse, multiple sexual partners, condom use (no) and birth control pill use (no) (between-group comparisons: all *p* < 0.05).

**Table 3:** Distribution of suicide-attempt-associated health-risk behaviors among the four physical fighting/sexual violence groups.

Risk behavior^a^	All	Physical fighting/sexual violence groups				*p* values for pair-wise comparisons ^b^					
		–/– (Group 1)	–/+(Group 2)	+ /– (Group 3)	+/+ (Group 4)	Group 1 vs 2	Group 1 vs 3	Group 1 vs 4	Group 2 vs 3	Group 2 vs 4	Group 3 vs 4
Total sample size	1755	827	360	318	250						
Seat belt use, never/rarely	171 (10.2)	55 (7.0)	33 (9.6)	30 (9.8)	53 (21.8)	0.1315	0.1281	** < 0.0001**	0.9579	** < 0.0001**	**0.0001**
Riding with a drinking driver	366 (21.4)	131 (16.1)	71 (20.5)	69 (22.2)	95 (39.9)	0.0675	0.0164	** < 0.0001**	0.6025	** < 0.0001**	** < 0.0001**
Weapon carrying	322 (19.0)	90 (11.0)	47 (13.6)	94 (30.1)	91 (41.0)	0.2132	** < 0.0001**	** < 0.0001**	** < 0.0001**	** < 0.0001**	0.0093
Weapon carrying at school	72 (4.3)	15 (1.9)	11 (3.2)	10 (3.2)	36 (15.3)	0.1837	0.1734	** < 0.0001**	0.9503	** < 0.0001**	** < 0.0001**
Gun carrying past 12 mos	75 (5.1)	10 (1.4)	9 (3.1)	22 (7.9)	34 (18.3)	0.0772	** < 0.0001**	** < 0.0001**	0.0098	** < 0.0001**	**0.0008**
Safety concerns at school	295 (16.8)	78 (9.4)	76 (21.2)	52 (16.4)	89 (35.7)	** < 0.0001**	**0.0009**	** < 0.0001**	0.1101	**0.0001**	** < 0.0001**
Threatened at school	270 (15.5)	65 (7.9)	52 (14.5)	68 (21.6)	85 (34.3)	**0.0005**	** < 0.0001**	** < 0.0001**	0.0169	** < 0.0001**	**0.0008**
Physical dating violence	209 (12.3)	31 (3.9)	71 (20.5)	25 (8.2)	82 (34.0)	** < 0.0001**	**0.0033**	** < 0.0001**	** < 0.0001**	**0.0002**	** < 0.0001**
Bullying at school	705 (40.6)	245 (29.8)	180 (50.6)	133 (42.1)	147 (60.5)	** < 0.0001**	**0.0001**	** < 0.0001**	0.0280	0.0165	** < 0.0001**
Electronic bullying	581 (33.3)	183 (22.2)	157 (43.9)	107 (33.6)	134 (55.4)	** < 0.0001**	**0.0001**	** < 0.0001**	**0.0066**	**0.0056**	** < 0.0001**
Ever cigarette use	545 (36.0)	177 (24.3)	124 (40.3)	117 (42.4)	127 (63.8)	** < 0.0001**	** < 0.0001**	** < 0.0001**	0.6014	** < 0.0001**	** < 0.0001**
Initiation of cigarette smoking, ≤ 12 years old	214 (12.6)	52 (6.4)	45 (13.0)	48 (15.7)	69 (30.4)	**0.0002**	** < 0.0001**	** < 0.0001**	0.3276	** < 0.0001**	**0.0001**
Current cigarette use	168 (11.2)	44 (6.1)	39 (12.8)	28 (10.0)	57 (28.8)	**0.0003**	0.0295	** < 0.0001**	0.2974	** < 0.0001**	** < 0.0001**
Smoked > 10 cigarettes	16 (1.0)	2 (0.2)	2 (0.6)	1 (0.3)	11 (4.9)	0.3772	0.8139	** < 0.0001**	0.6373	**0.0009**	**0.0006**
Electronic vapor product use	1121 (65.3)	448 (54.8)	259 (73.8)	221 (70.8)	193 (81.4)	** < 0.0001**	** < 0.0001**	** < 0.0001**	0.3954	0.0310	**0.0043**
Current electronic vapor product use	725 (44.2)	261 (33.2)	172 (51.0)	149 (50.3)	143 (63.8)	** < 0.0001**	** < 0.0001**	** < 0.0001**	0.8603	**0.0028**	**0.0021**
Current smokeless tobacco use	75 (4.4)	10 (1.2)	10 (2.8)	21 (6.9)	34 (14.3)	0.0522	** < 0.0001**	** < 0.0001**	0.0155	** < 0.0001**	**0.0042**
Current cigar use	138 (8.1)	27 (3.3)	26 (7.4)	37 (12.2)	48 (20.4)	**0.0024**	** < 0.0001**	** < 0.0001**	0.0379	** < 0.0001**	0.0096
Initiation of alcohol use, ≤ 12 years old	426 (24.9)	119 (14.7)	85 (24.0)	105 (34.2)	117 (48.3)	**0.0001**	** < 0.0001**	** < 0.0001**	**0.0039**	** < 0.0001**	**0.0008**
Current alcohol use	657 (40.8)	222 (28.6)	157 (48.0)	132 (45.7)	146 (66.1)	** < 0.0001**	** < 0.0001**	** < 0.0001**	0.5618	** < 0.0001**	** < 0.0001**
Current binge drinking	300 (18.7)	96 (12.2)	69 (21.5)	58 (20.1)	77 (37.4)	**0.0001**	**0.0010**	** < 0.0001**	0.6649	**0.0001**	** < 0.0001**
Largest number of drinks, ≥ 10 drinks	64 (4.8)	14 (2.1)	13 (5.0)	11 (4.6)	26 (15.4)	0.0215	0.0478	** < 0.0001**	0.8426	**0.0002**	**0.0002**
Ever marijuana use	807 (54.1)	311 (43.3)	185 (60.1)	162 (59.8)	149 (76.8)	** < 0.0001**	** < 0.0001**	** < 0.0001**	0.9441	**0.0001**	**0.0001**
Initiation of marijuana use, ≤ 12 years old	169 (9.8)	40 (4.9)	29 (8.2)	41 (13.4)	59 (24.4)	0.0268	** < 0.0001**	** < 0.0001**	0.0294	** < 0.0001**	**0.0009**
Current marijuana use	582 (33.8)	217 (26.5)	126 (35.5)	110 (35.5)	129 (54.0)	**0.0018**	**0.0030**	** < 0.0001**	0.9981	** < 0.0001**	** < 0.0001**
Ever synthetic marijuana use	212 (12.5)	55 (6.8)	40 (11.7)	42 (13.6)	75 (32.3)	**0.0060**	**0.0003**	** < 0.0001**	0.4665	** < 0.0001**	** < 0.0001**
Ever prescription pain medicine use	511 (29.5)	164 (20.0)	123 (34.3)	103 (33.1)	121 (50.2)	** < 0.0001**	** < 0.0001**	** < 0.0001**	0.7550	**0.0001**	**0.0001**
Ever cocaine use	132 (7.7)	28 (3.4)	24 (6.9)	24 (7.7)	56 (24.7)	0.0090	**0.0021**	** < 0.0001**	0.6707	** < 0.0001**	** < 0.0001**
Ever inhalant use	244 (14.4)	65 (8.1)	61 (17.4)	42 (13.9)	76 (32.3)	** < 0.0001**	**0.0035**	** < 0.0001**	0.2190	** < 0.0001**	** < 0.0001**
Ever heroin use	52 (3.0)	7 (0.9)	7 (2.0)	6 (1.9)	32 (13.6)	0.0946	0.1367	** < 0.0001**	0.9137	** < 0.0001**	** < 0.0001**
Ever methamphetamine use	74 (4.4)	10 (1.2)	15 (4.4)	9 (2.9)	40 (17.1)	**0.0008**	0.0527	** < 0.0001**	0.3153	** < 0.0001**	** < 0.0001**
Ever ecstasy use	121 (7.1)	25 (3.1)	17 (5.0)	34 (10.9)	45 (19.3)	0.1180	** < 0.0001**	** < 0.0001**	**0.0046**	** < 0.0001**	**0.0060**
Ever steroid use	53 (3.6)	5 (0.7)	14 (4.7)	5 (1.8)	29 (15.6)	** < 0.0001**	0.1163	** < 0.0001**	0.0516	** < 0.0001**	** < 0.0001**
Illegal injected drug use	39 (2.6)	4 (0.6)	8 (2.7)	4 (1.4)	23 (12.4)	**0.0039**	0.1628	** < 0.0001**	0.2846	** < 0.0001**	** < 0.0001**
Ever sexual intercourse	719 (49.4)	248 (35.7)	182 (62.3)	135 (49.6)	154 (79.0)	** < 0.0001**	**0.0001**	** < 0.0001**	**0.0024**	**0.0001**	** < 0.0001**
Sex before 13 years	101 (6.3)	14 (1.8)	28 (8.6)	13 (4.5)	46 (20.7)	** < 0.0001**	0.0143	** < 0.0001**	0.0391	** < 0.0001**	** < 0.0001**
Multiple sex partners	202 (12.5)	35 (4.5)	66 (20.1)	34 (11.7)	67 (29.9)	** < 0.0001**	** < 0.0001**	** < 0.0001**	**0.0044**	0.0083	** < 0.0001**
Current sexual activity, ≥ 1 people	574 (35.5)	207 (26.8)	133 (40.5)	102 (35.2)	132 (58.9)	** < 0.0001**	**0.0073**	** < 0.0001**	0.1694	** < 0.0001**	** < 0.0001**
Alcohol/drugs and sex	196 (12.1)	49 (6.3)	47 (14.3)	31 (10.5)	69 (31.5)	** < 0.0001**	0.0198	** < 0.0001**	0.1589	** < 0.0001**	** < 0.0001**
Condom use, no	407 (25.4)	118 (15.4)	122 (37.4)	67 (23.0)	100 (45.5)	** < 0.0001**	**0.0035**	** < 0.0001**	**0.0001**	0.0609	** < 0.0001**
Birth control pill use, no	147 (9.3)	48 (6.3)	46 (14.3)	15 (5.3)	38 (17.8)	** < 0.0001**	0.5213	** < 0.0001**	**0.0002**	0.2790	** < 0.0001**
Breakfast eating, 0 days	349 (23.3)	152 (21.0)	75 (25.2)	65 (23.1)	57 (29.2)	0.1477	0.4663	0.0153	0.5674	0.3191	0.1339
**Cumulative number of risk factors**											
Median (Q1, Q3)	6 (3, 11)	4 (2, 7)	8 (5, 11)	7 (4, 11)	13 (8, 17)	** < 0.0001**	** < 0.0001**	** < 0.0001**	0.2800	** < 0.0001**	** < 0.0001**
Min, max	0, 40	0, 22	0, 36	0, 26	0, 40						

^a^Risk factors that significantly associated with past- year suicide attempt. The full description for the question and response classification were presented in Supplementary Table 1.

^b^Bold values were statistically significant after a Bonferroni correction (αʹ = 0.05/6 = 0.0083).

**Figure 2:**
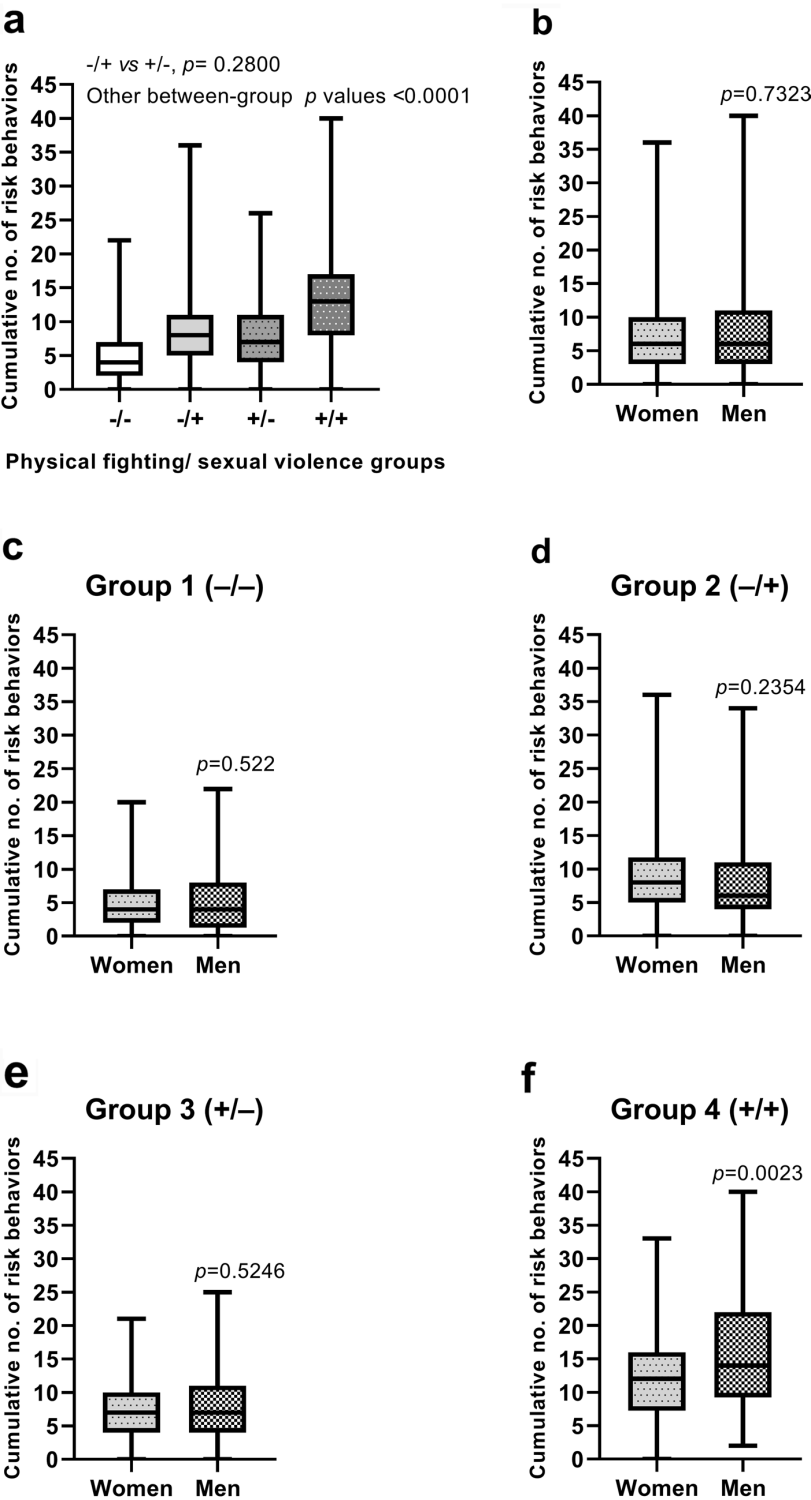
Cumulative number of suicide-attempt-associated health-risk behaviors among adolescents who reported having seriously considered attempting suicide during the past year, according to physical fighting/sexual violence group and sex. The analysis included the 42 suicide-attempt-associated health-risk behaviors (see Table 3).

The cumulative number of risk factors was significantly higher in the (physical fighting +/sexual violence –) group (median = 8, range: 0–36) and the (physical fighting –/sexual violence +) group (median = 7, range: 0–26) than that in the (physical fighting –/sexual violence –) group (median 4, range: 0–22); the cumulative number of risk factors was highest in the (physical fighting +/sexual violence +) group (median = 13, range: 0–40) (Table 3). The cumulative number of risk behaviors for men and women is shown in Fig. 2. Among the (physical fighting +/sexual violence +) group, men reported more risk behaviors (median = 14, range: 2–40) than women (median = 12, range: 0–33) (*p* = 0.0023). The differences in the number of risk behaviors between the two sexes in the other physical fighting/sexual violence groups were not statistically significant (all *p* > 0.05).

## Discussion

The concurrence of physical fighting and sexual violence was significantly associated with an increased risk of attempted suicide and attempted suicide requiring medical intervention among adolescent suicide ideators. However, the association pattern differed between men and women: among women, the associations between physical fighting and/or sexual violence and suicide behaviors were more likely to be statistically significant; in men, the concurrence of physical fighting and sexual violence was associated with a remarkably higher risk of attempted suicide requiring medical intervention. High rates of suicide-attempt-associated health-risk behaviors as well as a high cumulative number of health-risk behaviors were determined among the groups with concurrent physical fighting and sexual violence, especially among men.

Physical fighting and sexual violence are well-established risk factors/predictors for suicidal behaviors among adolescents and adults^12–15^. A previous study based on YRBS data showed that high school students who reported having attempted suicide during the past 12 months were more likely to also have reported fighting than were those who reported not having attempted suicide (61.5% vs 30.3%)^15^. Stack et al. reported that involvement in physical fighting raised the odds of a suicide attempt by 2.18 times^13^. In another study, an experience of sexual violence was shown to be associated with an increased risk of attempted suicide among high school men and women students (odds ratio = 11.3 and 4.54, respectively; *p* < 0.05)^14^. Consistent with previous findings, our analyses showed that both physical fighting and sexual violence were associated with an increased risk of attempted suicide. The potential role of both factors in the suicidal behavior trajectory can be understood in the context of the interpersonal theory of suicide^2^, according to which three constructs, thwarted belongingness, perceived burdensomeness, and the acquired capability for suicide, are needed for an individual to attempt suicide. The capability for suicide can be acquired by an increased tolerance of physical pain and a reduced fear of death through habituation to the physically painful and fearful aspects of self-harm^2^. Accordingly, physical fighting may act as a risk factor for suicidal behaviors, by increasing the individual’s acquired capability for suicide. In support of this explanation, individuals who reported engaging in physical fights had higher acquired capability scores^22^. The experience of sexual violence might lead to suicidal desires by increasing the victim’s sense of perceived burdensomeness and thwarted belongingness; it may also increase his or her capability for suicide, by increasing habituation to pain and fear^14^.

Our analyses showed that among the four study groups (physical fighting/sexual violence: –/–, –/+, +/–, and +/+), the group positive for both physical fighting and sexual violence had the highest risk of attempted suicide (adRR = 1.99, 95% CI 1.72, 2.29), suicide plan (adRR = 1.21, 95% CI 1.10, 1.32), and attempted suicide requiring medical intervention (adRR = 4.07, 95% CI 2.84, 5.85). Notably, the concurrence of physical fighting and sexual violence raised the risk of attempted suicide requiring medical intervention by four-fold, suggesting an association of this combined history with more severe forms of attempted suicide. This finding suggests that, in the search for factors predicting attempted suicide among ideators, the effects of clustered risk factors should be considered. Our results also emphasize the need for both targeted prevention and intervention efforts in adolescents with combined history of physical fighting and sexual violence.

This study identified associations between physical fighting/sexual violence and a wide variety of health-risk behaviors, many of which have been reported in previous studies^17,19,23,24^. For example, carrying a weapon was found to be associated with increased involvement in physical fighting^19^; early alcohol use and heavy drinking were shown to be associated with both fighting and suicide attempts among adolescents^24^; and a history of adolescent sexual victimization was found to be associated with increased likelihood of cigarette smoking, marijuana use, multiple sexual partners, and early sexual intercourse among college women^17^. According to problem behavior theory, risk-health behaviors might cluster together because they serve similar psychological and social development functions^20^. The risk behaviors investigated herein were selected from factors associated with the risk of attempted suicide. It should be noted that, because the YRBS questionnaire queries risk behaviors during the year prior to the survey, it may have missed common risk factors that manifested prior to the surveyed year that in turn increased the risk of past-year physical fighting, sexual violence, attempted suicide and related behaviors, and/or other risk behaviors. Also, as ours was a cross-sectional study, and the interactions between multiple past and present risk factors are likely to be complex, establishing causality between the observed associations of suicide behaviors with other risk behaviors was not possible. Considering the complexity of the pathways of suicide behaviors, isolating the effect of a single factor might be unrealistic, and interventions targeting single factors might not suffice to prevent suicide attempts. Moreover, the acquired capability for suicide might accumulate through multiple risk factors, with amplified capability resulting in more lethal suicide behaviors. Therefore, in the development of intervention plans, it is important to identify the key factors that lead to subsequent risk behaviors and the reversible factors that can serve as intervention targets.

Among both sexes, the concurrence of physical fighting and sexual violence was associated with a high risk for attempted suicide requiring medical intervention. The latter was reported in 26.5% of women and 32.8% of men. The adRR for attempted suicide requiring medical intervention was remarkably higher among men (adRR = 6.25, 95% CI 3.32, 12.28) than women (adRR = 3.33, 95% CI 2.14, 5.17), using the sex-specific risk in the (physical fighting –/sexual violence –) group as the reference. The sex difference in suicidal behaviors is well known. In most of the world, women are more likely than men to attempt suicide, but they are less likely to die by suicide^16^. An explanation from the interpersonal theory of suicide is that women are less likely to develop an acquired capability for suicidal behaviors because they generally experience fewer events that could reduce their fear of self-injury through habituation (e.g., exposure to guns, physical fights, violent sports), and they have less pain and fear tolerance than men^2^. Our finding supports this explanation in that, among adolescents with concurrent physical fighting and sexual violence, men reported more health-risk behaviors than women (man: median = 14, range: 2–40; women: median = 12, range: 0–33; *p* = 0.0023). It also provides insights into the sex difference in suicidal behaviors while further highlighting the importance of examining an individual’s risk factor profile when evaluating the suicide risk.

The strengths of this study include its novel investigation of (i) the association between concurrent physical fighting and sexual violence and the risk of attempted suicide among adolescent suicide ideators and (ii) the clustering of suicide-attempt-associated health-risk behaviors among several sub-populations. In addition, in estimating the strength of the associations between risk factors and suicidal behaviors, unlike studies that used logistic regression to estimate the odds ratio, our study employed a log binomial regression to estimate the RR and AR, which are both more intuitive and more easily interpreted^25^.

This study had several limitations. First, its cross-sectional design limited our ability to make causal inferences. For example, it is possible that some of the reported experiences of physical fighting and sexual violence occurred after the attempted suicide. Also, as discussed above, there may have been other common risk factors that increased the risk of physical fighting, sexual violence, and suicidal behaviors, such that whether physical fighting and sexual violence had an additive or cumulative effect on attempted suicide could not be determined. Nevertheless, the identified patterns are informative in understanding how suicide-related risk behaviors cluster and for identifying high-risk populations needing multiple behavioral interventions. Longitudinal studies that test the causality between sexual violence, physical fighting, suicide behaviors, and other risk behaviors are still needed. Second, only past-year physical fights and past-year sexual violence were investigated. However, the impact of sexual violence can be long-lasting. If sexual violence occurred previous to the past year, it would not have been taken into account and those respondents would have been assigned to the reference group. This misclassification could have affected the results of our study. Third, the YRBS data were collected from self-reported questionnaires, such that the answers were subject to recall bias and to classification error arising from individual differences in understanding the queries. Fourth, because the YRBS is a school-based survey it may not be representative of adolescents who no longer attend school. Lastly, the data were obtained from adolescents in the United States; the combined effects may be different under different cultural and social-developmental conditions^26^. Thus, our findings remain to be validated in different countries and populations. Both a better understanding of suicidal behaviors and the design of effective interventions will require further research.

To conclude, the concurrence of physical fighting and sexual violence among adolescent suicide ideators was shown to be significantly associated with an increased risk of attempted suicide and attempted suicide requiring medical intervention. Several different health-risk behaviors were shown to cluster with the concurrence of physical fighting and sexual violence. This was especially the case among men, consistent with their higher rate of attempted suicide requiring medical intervention. Further studies are needed to explore the mechanism governing the interaction of multiple risk factors in suicide pathways. The development of effective suicide intervention strategies awaits a determination of the key factors that lead to risk behaviors as well as the identification of reversible factors that could serve as intervention targets.

## Supplementary Information

Supplementary Information.

## Data Availability

The datasets generated during and/or analyzed for this study are available at: https://www.cdc.gov/healthyyouth/data/yrbs/data.htm.
