# Supplementary material for: The concurrence of sexual violence and physical fighting among adolescent suicide ideators and the risk of attempted suicide
Source: Sci Rep. 2022 Mar 28;12:5290. doi: 10.1038/s41598-022-09387-3 (PMC8964683; doi:10.1038/s41598-022-09387-3)
Supplement: Supplementary file 1 — Supplementary Information. [file 41598_2022_9387_MOESM1_ESM.docx]

**Supplementary material**





**Supplementary Figure 1.** Flow diagram of study population

**Supplementary Table 1**. Distribution of health-risk behaviors among adolescents who reported having seriously considered attempting suicide during the past year, as reported in the 2019 YRBS (n=2095)

| No. | Variable label | Question | Responses that classified as a risk behavior | Total | Attempt suicide | | |
| --- | --- | --- | --- | --- | --- | --- | --- |
|  |  |  |  |  | No | Yes | *p* |
| Q8 | Seat belt use | How often do you wear a seat belt when riding in a car driven by someone else? | Never/rarely | 207 (10.3) | 95 ( 8.0) | 112 (13.7) | 0.0001 |
| Q9 | Riding with a drinking driver | During the past 30 days, how many times did you ride in a car or other vehicle driven by someone who had been drinking alcohol? | ≥1 times | 427 (21.8) | 223 (19.3) | 204 (25.3) | 0.0018 |
| Q10 | Drinking and driving | During the past 30 days, how many times did you drive a car or other vehicle when you had been drinking alcohol? | ≥1 times | 87 ( 4.5) | 50 ( 4.3) | 37 ( 4.9) | 0.5759 |
| Q11 | Texting and driving | During the past 30 days, on how many days did you text or e-mail while driving a car or other vehicle? | ≥1 days | 447 (24.0) | 274 (24.6) | 173 (23.1) | 0.4723 |
| Q12 | Weapon carrying | During the past 30 days, on how many days did you carry a weapon such as a gun, knife, or club? | ≥1 days | 371 (19.6) | 181 (16.3) | 190 (24.3) | <0.0001 |
| Q13 | Weapon carrying at school | During the past 30 days, on how many days did you carry a weapon such as a gun, knife, or club on school property? | ≥1 days | 89 ( 4.5) | 37 ( 3.2) | 52 ( 6.5) | 0.0008 |
| Q14 | Gun carrying past 12 mos | During the past 12 months, on how many days did you carry a gun? (Do not count the days when you carried a gun only for hunting or for a sport, such as target shooting.) | ≥1 days | 89 ( 5.6) | 35 ( 3.8) | 54 ( 8.2) | 0.0002 |
| Q15 | Safety concerns at school | During the past 30 days, on how many days did you not go to school because you felt you would be unsafe at school or on your way to or from school? | ≥1 days | 357 (17.1) | 169 (13.7) | 188 (22.0) | <0.0001 |
| Q16 | Threatened at school | During the past 12 months, how many times has someone threatened or injured you with a weapon such as a gun, knife, or club on school property? | ≥1 times | 326 (15.7) | 156 (12.7) | 170 (20.1) | <0.0001 |
| Q22 | Physical dating violence | During the past 12 months, how many times did someone you were dating or going out with physically hurt you on purpose? (Count such things as being hit, slammed into something, or injured with an object or weapon.) | ≥1 times | 252 (12.5) | 99 ( 8.3) | 153 (18.8) | <0.0001 |
| Q23 | Bullying at school | During the past 12 months, have you ever been bullied on school property? | Yes | 849 (41.1) | 439 (35.9) | 410 (48.6) | <0.0001 |
| Q24 | Electronic bullying | During the past 12 months, have you ever been electronically bullied? (Count being bullied through texting, Instagram, Facebook, or other social media.) | Yes | 701 (33.8) | 348 (28.4) | 353 (41.5) | <0.0001 |
| Q30 | Ever cigarette use | Have you ever tried cigarette smoking, even one or two puffs? | Yes | 636 (36.4) | 334 (32.3) | 302 (42.4) | <0.0001 |
| Q31 | Initiation of cigarette smoking | How old were you when you first tried cigarette smoking, even one or two puffs? | ≤12 years old | 249 (13.3) | 114 (10.3) | 135 (17.6) | <0.0001 |
| Q32 | Current cigarette use | During the past 30 days, on how many days did you smoke cigarettes? | ≥1 days | 206 (11.4) | 87 ( 8.1) | 119 (16.2) | <0.0001 |
| Q33 | Smoked >10 cigarettes | During the past 30 days, on the days you smoked, how many cigarettes did you smoke per day? | >10 cigarettes | 17 ( 0.9) | 1 ( 0.1) | 16 ( 2.0) | <0.0001 |
| Q34 | Electronic vapor product use | Have you ever used an electronic vapor product? | Yes | 1326 (64.9) | 752 (62.0) | 574 (69.2) | 0.0008 |
| Q35 | Current electronic vapor product use | During the past 30 days, on how many days did you use an electronic vapor product? | ≥1 days | 879 (44.9) | 485 (41.3) | 394 (50.2) | 0.0001 |
| Q37 | Current smokeless tobacco use | During the past 30 days, on how many days did you use chewing tobacco, snuff, dip, snus, or dissolvable tobacco products, such as Copenhagen, Grizzly, Skoal, or Camel Snus? (Do not count any electronic vapor products.) | ≥1 days | 99 ( 5.0) | 43 ( 3.7) | 56 ( 7.0) | 0.0015 |
| Q38 | Current cigar use | During the past 30 days, on how many days did you smoke cigars, cigarillos, or little cigars? | ≥1 days | 164 ( 8.4) | 66 ( 5.7) | 98 (12.3) | <0.0001 |
| Q39 | All tobacco product cessation | During the past 12 months, did you ever try to quit using all tobacco products, including cigarettes, cigars, smokeless tobacco, shisha or hookah tobacco, and electronic vapor products? | Yes | 334 (18.5) | 174 (16.3) | 160 (21.7) | 0.0046 |
| Q40 | Initiation of alcohol use | How old were you when you had your first drink of alcohol other than a few sips? | ≤12 years old | 488 (23.9) | 242 (20.1) | 246 (29.4) | <0.0001 |
| Q41 | Current alcohol use | During the past 30 days, on how many days did you have at least one drink of alcohol? | ≥1 days | 777 (40.4) | 434 (37.4) | 343 (44.8) | 0.0013 |
| Q42 | Current binge drinking | During the past 30 days, on how many days did you have 4 or more drinks of alcohol in a row, that is, within a couple of hours (if you are female) or 5 or more drinks of alcohol in a row, that is, within a couple of hours (if you are male)? | ≥1 days | 343 (19.0) | 187 (17.4) | 156 (21.4) | 0.0378 |
| Q43 | Largest number of drinks | During the past 30 days, what is the largest number of alcoholic drinks you had in a row, that is, within a couple of hours? | ≥10 drinks | 70 ( 4.5) | 29 ( 3.1) | 41 ( 6.7) | 0.0011 |
| Q45 | Ever marijuana use | During your life, how many times have you used marijuana? | ≥1 times | 930 (53.7) | 515 (49.9) | 415 (59.3) | 0.0001 |
| Q46 | Initiation of marijuana use | How old were you when you tried marijuana for the first time? | ≤12 years old | 210 (10.2) | 93 ( 7.6) | 117 (13.9) | <0.0001 |
| Q47 | Current marijuana use | During the past 30 days, how many times did you use marijuana? | ≥1 times | 702 (34.2) | 371 (30.5) | 331 (39.6) | <0.0001 |
| Q48 | Ever synthetic marijuana use | During your life, how many times have you used synthetic marijuana? | ≥1 times | 252 (13.2) | 116 (10.3) | 136 (17.3) | <0.0001 |
| Q49 | Ever prescription pain medicine use | During your life, how many times have you taken prescription pain medicine without a doctor's prescription or differently than how a doctor told you to use it? | ≥1 times | 589 (29.6) | 275 (23.3) | 314 (38.6) | <0.0001 |
| Q50 | Ever cocaine use | During your life, how many times have you used any form of cocaine, including powder, crack, or freebase? | ≥1 times | 151 ( 8.1) | 59 ( 5.4) | 92 (12.0) | <0.0001 |
| Q51 | Ever inhalant use | During your life, how many times have you sniffed glue, breathed the contents of aerosol spray cans, or inhaled any paints or sprays to get high? | ≥1 times | 292 (15.2) | 129 (11.3) | 163 (20.8) | <0.0001 |
| Q52 | Ever heroin use | During your life, how many times have you used heroin (also called smack, junk, or China White)? | ≥1 times | 71 ( 3.6) | 19 ( 1.7) | 52 ( 6.4) | <0.0001 |
| Q53 | Ever methamphetamine use | During your life, how many times have you used methamphetamines (also called speed, crystal meth, crank, ice, or meth)? | ≥1 times | 90 ( 4.6) | 29 ( 2.5) | 61 ( 7.6) | <0.0001 |
| Q54 | Ever ecstasy use | During your life, how many times have you used ecstasy (also called MDMA)? | ≥1 times | 142 ( 7.4) | 56 ( 5.0) | 86 (10.9) | <0.0001 |
| Q55 | Ever steroid use | During your life, how many times have you taken steroid pills or shots without a doctor's prescription? | ≥1 times | 62 ( 3.9) | 17 ( 1.8) | 45 ( 6.8) | <0.0001 |
| Q56 | Illegal injected drug use | During your life, how many times have you used a needle to inject any illegal drug into your body? | ≥1 times | 43 ( 2.7) | 7 ( 0.7) | 36 ( 5.4) | <0.0001 |
| Q57 | Illegal drugs at school | During the past 12 months, has anyone offered, sold, or given you an illegal drug on school property? | Yes | 1308 (64.1) | 796 (65.5) | 512 (62.1) | 0.1209 |
| Q58 | Ever sexual intercourse | Have you ever had sexual intercourse? | Yes | 840 (50.0) | 440 (43.6) | 400 (59.6) | <0.0001 |
| Q59 | Sex before 13 years | How old were you when you had sexual intercourse for the first time? | ≤12 years old | 108 ( 5.8) | 41 ( 3.7) | 67 ( 9.1) | <0.0001 |
| Q60 | Multiple sex partners | During your life, with how many people have you had sexual intercourse? | ≥4 people | 229 (12.3) | 102 ( 9.1) | 127 (17.2) | <0.0001 |
| Q61 | Current sexual activity | During the past 3 months, with how many people did you have sexual intercourse? | ≥1 people | 672 (36.1) | 353 (31.5) | 319 (43.3) | <0.0001 |
| Q62 | Alcohol/drugs and sex | Did you drink alcohol or use drugs before you had sexual intercourse the last time? | Yes | 225 (12.4) | 108 ( 9.9) | 117 (16.1) | 0.0001 |
| Q63 | Condom use | The last time you had sexual intercourse, did you or your partner use a condom? | No | 465 (25.2) | 237 (21.2) | 228 (31.3) | <0.0001 |
| Q64 | Birth control pill use | The last time you had sexual intercourse, what one method did you or your partner use to prevent pregnancy? | No method was used to prevent pregnancy | 161 ( 8.9) | 68 ( 6.2) | 93 (13.0) | <0.0001 |
| Q69 | Fruit juice drinking | During the past 7 days, how many times did you drink 100% fruit juices such as orange juice, apple juice, or grape juice? (Do not count punch, Kool-Aid, sports drinks, or other fruit-flavored drinks.) | None | 634 (32.6) | 390 (33.9) | 244 (30.8) | 0.1537 |
| Q70 | Fruit eating | During the past 7 days, how many times did you eat fruit? (Do not count fruit juice.) | None | 275 (13.8) | 151 (12.8) | 124 (15.4) | 0.1124 |
| Q71 | Green salad eating | During the past 7 days, how many times did you eat green salad? | None | 819 (44.8) | 488 (45.3) | 331 (44.0) | 0.5996 |
| Q72 | Potato eating | During the past 7 days, how many times did you eat potatoes? (Do not count french fries, fried potatoes, or potato chips.) | None | 712 (38.9) | 430 (39.9) | 282 (37.5) | 0.3063 |
| Q73 | Carrot eating | During the past 7 days, how many times did you eat carrots? | None | 1029 (56.4) | 586 (54.6) | 443 (59.1) | 0.0613 |
| Q74 | Other vegetable eating | During the past 7 days, how many times did you eat other vegetables? (Do not count green salad, potatoes, or carrots.) | None | 375 (20.6) | 207 (19.3) | 168 (22.4) | 0.112 |
| Q75 | Soda drinking | During the past 7 days, how many times did you drink a can, bottle, or glass of soda or pop, such as Coke, Pepsi, or Sprite? (Do not count diet soda or diet pop.) | ≥1 times | 1241 (73.6) | 712 (71.9) | 529 (76.1) | 0.0563 |
| Q76 | No milk drinking | During the past 7 days, how many glasses of milk did you drink? (Count the milk you drank in a | None | 567 (33.8) | 340 (34.1) | 227 (33.3) | 0.7526 |
| Q77 | Breakfast eating | During the past 7 days, on how many days did you eat breakfast? | 0 days | 401 (23.3) | 213 (21.0) | 188 (26.6) | 0.0076 |
| Q78 | Physical activity >= 5 days | During the past 7 days, on how many days were you physically active for a total of at least 60 minutes per day? (Add up all the time you spent in any kind of physical activity that increased your heart rate and made you breathe hard some of the time.) | ≤4 days | 1376 (67.3) | 817 (67.2) | 559 (67.3) | 1 |
| Q79 | Television watching | On an average school day, how many hours do you watch TV? | ≥ 3 hours per day | 440 (22.4) | 244 (20.9) | 196 (24.6) | 0.054 |
| Q80 | How many hours/day play video games | On an average school day, how many hours do you play video or computer games or use a computer for something that is not school work? (Count time spent playing games, watching videos, texting, or using social media on your smartphone, computer, Xbox, PlayStation, iPad, or other tablet.) | ≥ 3 hours per day | 1107 (54.3) | 657 (54.2) | 450 (54.3) | 0.9639 |
| Q81 | PE attendance | In an average week when you are in school, on how many days do you go to physical education (PE) classes? | 0 days | 933 (55.8) | 549 (55.6) | 384 (56.1) | 0.8413 |
| Q82 | Sports team participation | During the past 12 months, on how many sports teams did you play? (Count any teams run by your school or community groups.) | 0 teams | 897 (50.5) | 534 (51.3) | 363 (49.4) | 0.4409 |
